# Implementing a system for the real-time risk assessment of patients considered for intensive care

**DOI:** 10.1186/s12911-020-01176-0

**Published:** 2020-07-16

**Authors:** Simarjot S. Dahella, James S. Briggs, Paul Coombes, Nazli Farajidavar, Paul Meredith, Timothy Bonnici, Julie L. Darbyshire, Peter J. Watkinson

**Affiliations:** 1 Centre for Healthcare Modelling and Informatics, University of Portsmouth, Buckingham Building, Lion Terrace, Portsmouth, PO1 3HE UK; 2 IM&T, John Radcliffe Hospital, Headley Way, Oxford, OX3 9DU UK; 3 Department of Engineering, Institute of Biomedical Engineering, University of Oxford, Old Road Campus Research Building, Oxford, OX3 7DQ UK; 4 Research & Innovation, Portsmouth Hospitals NHS Trust, Queen Alexandra Hospital, Portsmouth, PO6 3LY UK; 5 Critical Care Department, University College London NHS Foundation Trust, 235 Euston Road, London, NW1 2BU UK; 6 Nuffield Department of Clinical Neurosciences, University of Oxford, John Radcliffe Hospital, Headley Way, Oxford, OX3 9DU UK

**Keywords:** Early warning score, Vital signs, Pathology data, Portable format for analytics, PFA, Human-computer interaction, HCI, Information visualisation, Clinical decision making, OSGi

## Abstract

**Background:**

Delay in identifying deterioration in hospitalised patients is associated with delayed admission to an intensive care unit (ICU) and poor outcomes. For the HAVEN project (HICF ref.: HICF-R9–524), we have developed a mathematical model that identifies deterioration in hospitalised patients in real time and facilitates the intervention of an ICU outreach team. This paper describes the system that has been designed to implement the model. We have used innovative technologies such as Portable Format for Analytics (PFA) and Open Services Gateway initiative (OSGi) to define the predictive statistical model and implement the system respectively for greater configurability, reliability, and availability.

**Results:**

The HAVEN system has been deployed as part of a research project in the Oxford University Hospitals NHS Foundation Trust. The system has so far processed > 164,000 vital signs observations and > 68,000 laboratory results for > 12,500 patients and the algorithm generated score is being evaluated to review patients who are under consideration for transfer to ICU. No clinical decisions are being made based on output from the system. The HAVEN score has been computed using a PFA model for all these patients. The intent is that this score will be displayed on a graphical user interface for clinician review and response.

**Conclusions:**

The system uses a configurable PFA model to compute the HAVEN score which makes the system easily upgradable in terms of enhancing systems’ predictive capability. Further system enhancements are planned to handle new data sources and additional management screens.

## Background

Hospital inpatients whose condition deteriorates are often transferred from a general ward to an Intensive Care Unit (ICU) in order for them to receive a higher level of care. Such unplanned ICU admissions from within hospital typically make up over half of the total ICU admissions [1]. Unplanned ICU admission is associated with a poor patient outcome, but timely admission may improve that outcome [2]. It is imperative therefore to identify patients who would need ICU admission in time to avert an unplanned admission. Unplanned ICU admission can be effectively averted by using a system that has a good predictive model and stimulates situational awareness of the relevant clinicians by visualisation. Visualisation can facilitate good clinical decision-making processes [3].

To facilitate early recognition of deteriorating patients and to avoid unplanned ICU admission, we have designed and developed the “Hospital alerting via electronic noticeboard (HAVEN)” system. HAVEN is a decision support system that can be used hospital-wide. It uses a configurable predictive model and a user-defined graphical user interface to display details of patients at risk of unplanned admission to the ICU. This paper describes the architecture and technologies that we used to build the system.

The National Institute for Health and Care Excellence (NICE) has recommended the use of multiple-parameter or aggregate weighted scoring systems for monitoring acutely ill patients since 2007 [4]. Much research has been done in creating models for computing the early warning scores [5–7] and comparing the different models that compute the scores [8, 9]. It has been reported by NHS Digital, however, that recorded critical care periods in English hospitals have increased by 25% from 2010/11 to 2015/16 [1]. In 2015/16, more than half of these ICU admissions were ‘unplanned’ [10, 11]. Delays in admitting ward patients to ICU are common, with high short-term mortality because of the difficulty in triaging deteriorating patients in wards [12]. This is a global problem [13].

Another important aspect of outcome prediction in healthcare systems is the predictive model itself. Traditionally, a predictive model is static and embedded in the system [14]. Changing or updating the model in the system is usually a complicated task. This may mean that models are not updated as often as they should be. Hence, clinicians end up using systems that are not optimal.

We used innovative software technologies to enable rapid system updates. This is essential when the predictive model is frequently improved in its means of identifying accurately the sickest patients who will derive most benefit from increased care. The technologies used and details of the system build are described in the following sections.

### Implementation architecture

We explored technologies for developing the system in a modular way that can be easily deployed and dynamically updated. Additionally, we evaluated the techniques for defining and executing the predictive model. The model needed to be configurable so that it can be updated dynamically when system capabilities are enhanced.

HAVEN has been implemented as a web-based application. It is therefore accessible in any part of the hospital using any machine with an internet connection without installing any additional software beyond a standard web browser.

The technology stack we decided to use for the application implementation consists of the Java programming language [15], MySQL database [16], Open Services Gateway initiative (OSGi) Framework [17], PFA model [18], and ReactJS [19].

The OSGi specification describes a modular system and a service platform for the Java programming language that implements a complete and dynamic component model. Components are deployed as bundles in an OSGi container. Each bundle of the system is complete in itself in terms of its lifecycle management. The OSGi bundles hide their implementations from other bundles while communicating through services. So, OSGi provides a collaborative environment for micro-services to work together to fulfil the overall goal of the system. The dynamic component model makes the dynamic updating of bundles possible. Additionally, system components (bundles) can be remotely installed, started, stopped, and uninstalled without requiring a full system reboot [20]. The fact that no downtime is required for maintenance and enhancement of the system makes it a viable choice over traditional monolithic software development approaches.

MySQL 5.6 server has been used for the aggregated system database. It is an open-sourcerelational database management system (RDBMS). MySQL is easy to use, secure, scalable, and yet extremely powerful.

The system is designed to make the statistical model dynamically configurable. To achieve this, we have used the Portable Format for Analytics (PFA). PFA is a new standard for statistical models. It provides a way for the analytics team to describe and implement predictive models. The model can be provided to the deployment team for application in the production environment. A scoring model is an executable module that performs a purely mathematical task. It has a well-defined input and output. The HAVEN model is configured in the aggregated database which is then loaded by the system to compute the HAVEN score, which is generated from physiological data, laboratory test results, and other key variables collated for each patient.

Apart from micro-level deployable components which are deployed as bundles in an OSGi container, the system is conceptually and virtually divided into different layers. Different layers of the system are explained in the following sections, and components within each layer are shown in the system architecture diagram.

### Data aggregation

The patient-specific clinical and administrative data are sourced in real-time from different “live” hospital systems, and are integrated by the ‘HAVEN Integration Engine’ (HIE). Currently, the HAVEN system supports data from three clinical systems:

1. The Patient Administration System (PAS) for notification of Admissions, Discharges and Transfers (ADT);
2. The Vital Signs collection system (known as SEND - System for Electronic Notification and Documentation [21]); and
3. The hospital’s pathology laboratory system.

**Fig. 1:**
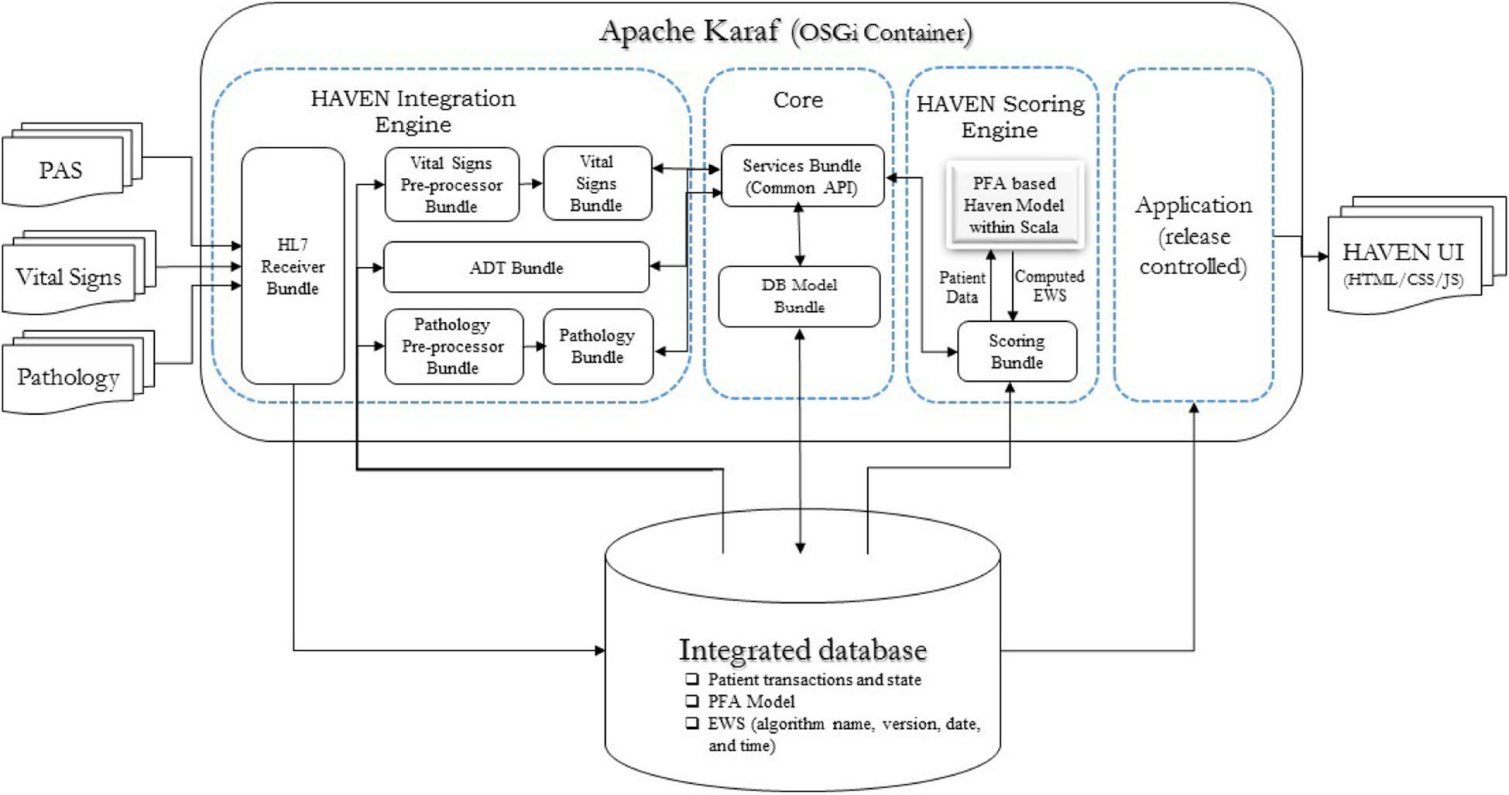
HAVEN Architecture Diagram showing the components and data flow

While the system has been configured to use data available within its trial environment, it has been designed to accept commonly available data from a variety of system sources. All coding of data within the clinical systems conforms to current, common terminology. Messages arrive in a standard HL7 version 2 format (used commonly across the NHS and worldwide). The architecture of the HAVEN system (Fig. 1) shows the HIE, deployed in an OSGi container, receiving data from different sources.

The HIE takes those inputs and processes the data for the ‘HAVEN Scoring Engine’ (HSE) to compute the HAVEN score for each patient. Each HL7 message contains a partial package of data that includes patient demographics, clinical physiology, and laboratory results. The HIE receives the incoming HL7 message, validates it against expected HL7 message structure, pre-processes the data items contained within it, and stores them in the integrated database. Abiding by the ‘Separation of Concerns’ design principle, each separate set of linked functionalities is implemented and bundled in a separate component which is then deployed in an OSGi container. There are separate components within the OSGi container that process each type of message, for example ADT messages, vital signs data, and laboratory results.

The ‘Core’ as shown in Fig. 1 contains the common code that is used by multiple other bundles. It encapsulates the database model and data access functionality via services.

### Scoring

The ‘Haven Scoring Engine’ computes the HAVEN Score for a patient based on the currently configured HAVEN predictive model (specified as a PFA document) that is stored in the integrated system database. Development and evaluation of the HAVEN risk score will be the subject of a future publication from the project team.

**Fig. 2:**
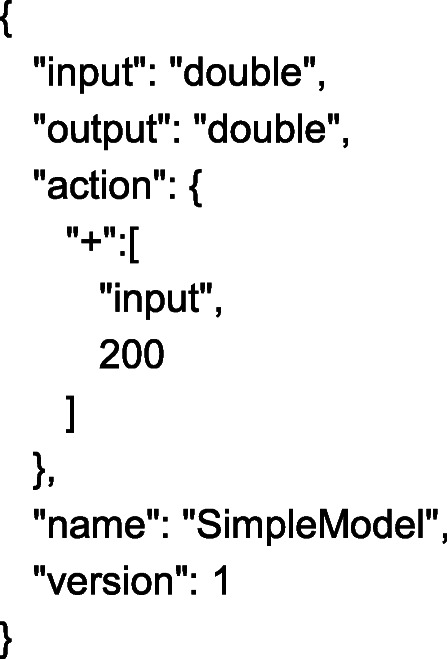
Example of PFA

PFA is an emerging standard for statistical models and data transformation engines [18]. A PFA document is a JSON format document with additional constraints. We also explored the use of Predictive Model Markup Language (PMML) - this is an XML-based language-neutral way to encode models, and is a well-established format in the analytics space. However, PMML has limited support for computation as it has only a standard defined set of supported models. Specific needs outside the standard require tremendous effort, or may not be possible. PFA provides the flexibility of arbitrary function composition. A very simple example of a PFA model is shown in Fig. 2 below. It just adds a number to the input and returns the result.

One could implement complex models (e.g. a clustering model, a tree model, a change detection model, a segmented model and many more) using PFA, with the possibility of extending the computational capabilities with customised functions.

Another benefit of using PFA is that the entire scoring flow is represented in a standardized manner in a single executable document. All the additional pre- and post-processing can be encapsulated by the analytics team in that document. No separate code fragment or script is required by the engineering team for model usage. This makes the operationalisation of the models “turn-key”. These features of PFA make it ideal for defining and executing the model to compute the HAVEN score.

**Fig. 3:**
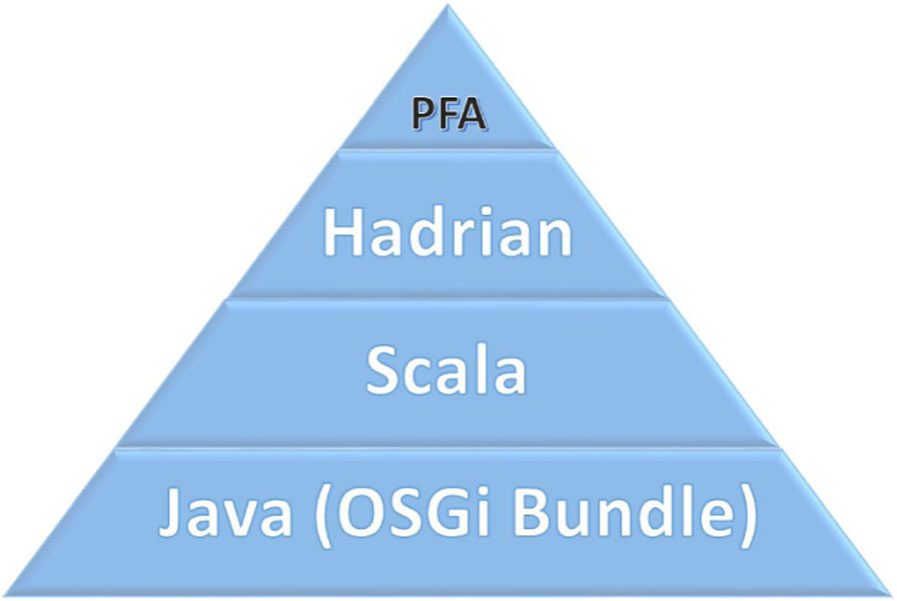
The technology stack used to execute the PFA model

The PFA model can be executed using the Hadrian library [22]. Hadrian is designed to be embedded in applications or used as a scoring engine container. The technology stack used to execute the PFA model in HAVEN system is shown in Fig. 3.

The Haven Scoring Engine is also deployed as a bundle in the OSGi container. Scala, a general-purpose programming language, has been embedded within the Haven Scoring Engine to provide language interoperability with Java and to embed the Hadrian API, which is capable of executing a PFA-based model.

On receiving the latest data for each patient, the system computes their HAVEN score using the PFA-based HAVEN model that has been configured. The system has the capability to compute multiple early warning scores based on differently configured PFA models.

### User interface

The HAVEN software is built as a web-based application to enable greater flexibility for deployment across multiple hardware platforms. The Spring-boot [23] framework has been used to implement the server side of the User Interface (UI), handling requests coming from the client’s browser. It uses a Rest API [24] with data in JSON [25] format. The data provided by the Rest API is subsequently used by the client-side implementation of the UI.

The client side of the UI is implemented in ReactJS [19], a JavaScript library. It allows the vital sign observation data to be pre-processed within the web-browser, thus minimising the number of requests made to the server. ReactJS enables the creation of large-scale web-applications that use dynamic data and can change over time without reloading the page, hence providing speed, simplicity, and scalability. It is used for building highly-dynamic and interactive user interfaces. The client-side implementation is then packaged using the JavaScript package manager, NPM [26].

The configurability of the system enables different user groups to easily view patient groups of interest from one system.

The UI facilitates data accessibility by providing user-customised views for carers, nursing staff, and clinicians. It summarises the patients’ recent status and represents the computed HAVEN score for each. It also provides the patients’ demographic overview along with an option to review the physiological data in detail. It focusses on summarising and visualising the data in a manner that suits a user group purpose.

The UI was developed in collaboration with the clinical teams. This ensured a user-centred approach in keeping with best user-design practice. Applied Cognitive Task Analysis interviews [27] and Card Sort tasks [28] were conducted with clinical staff. These and Process Mapping [29, 30] of workflows enabled a broader understanding of how variability of individual patient conditions can affect the whole team.

**Fig. 4:**
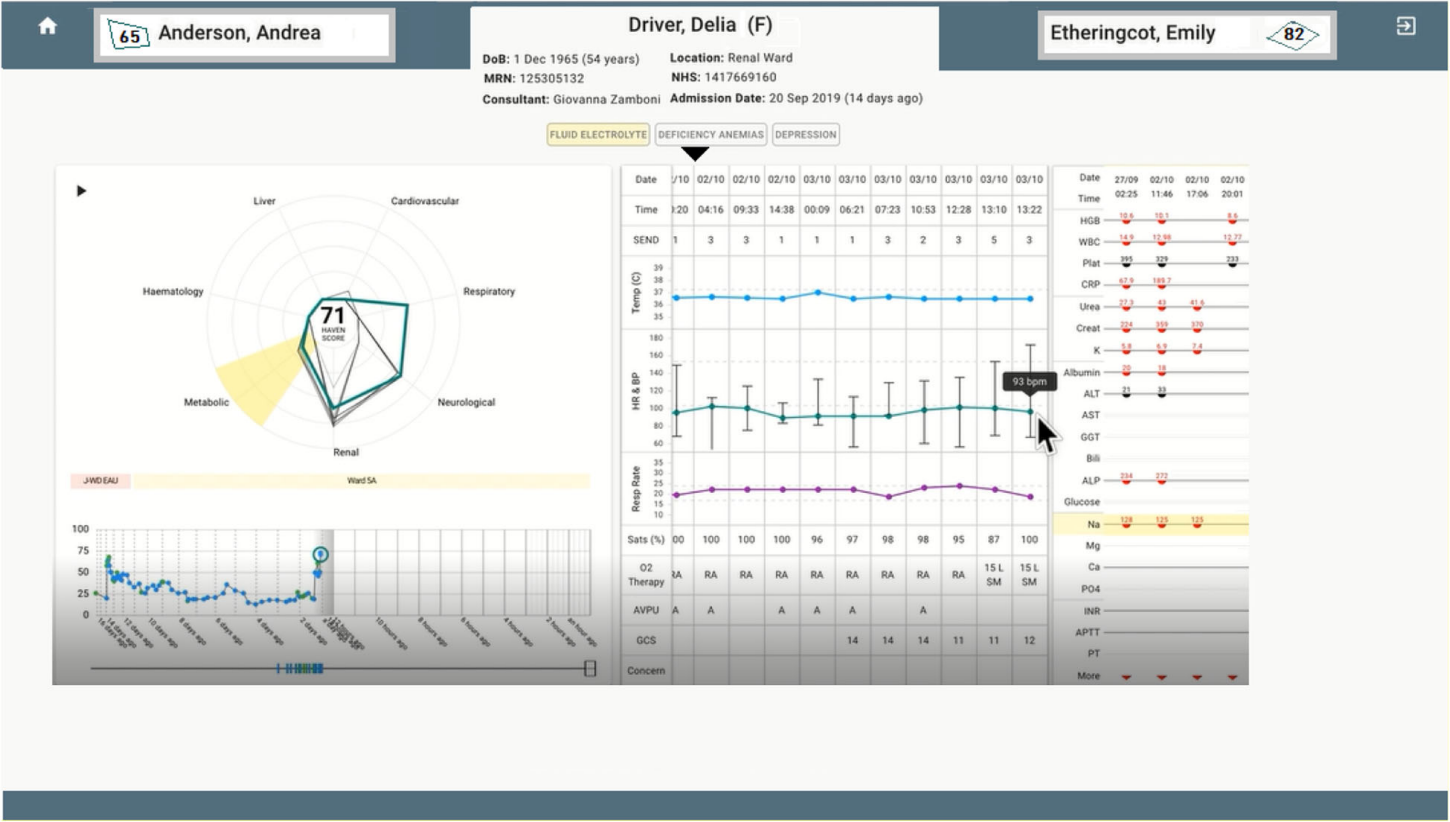
Screenshot of final UI (showing fictitious patient information)

The information from these activities guided decisions on both UI content and layout. The UI development was an iterative process. Each development stage was evaluated using standard human factors measures of usability, understanding, efficiency, and accuracy. The final version (see Fig. 4) performed well across all areas, resulting in a UI that affords accurate understanding with low levels of cognitive workload.

## Discussion

The system has been used as part of the HAVEN research project using patient data at Oxford University Hospitals NHS Foundation Trust. The system has so far processed > 164,000 vital signs observations and > 68,000 laboratory results for > 125,000 hospital inpatients and shown its capability to process large amounts of data effectively. It is being used as part of the HAVEN project’s evaluation phase to review the effectiveness of the system in identifying patients who might benefit from transfer to ICU. No clinical decisions have been made based on the output from the prototype system. All evaluations have been completed using retrospective data. The clinical and the user interface design parts of the project will be the subject of separate forthcoming publications.

We have demonstrated the technological benefits of our system architecture.

PFA is an emerging standard for describing the statistical model in data analysis and the statistical domain. It is usually executed within a Python or standalone Java application. HAVEN has included the PFA based statistical scoring model in its system. It is the first health-related software system we are aware of that uses the OSGi (Open Services Gateway initiative) Framework in combination with PFA. OSGi is a well-established technology and widely used by many renowned organisations. It replaces the traditional monolithic architecture software development approach with a new micro-services one.

The HAVEN system exploits the benefits of both technologies. By being able to execute the PFA model by embedding the required technologies/libraries like Scala and Hadrian in an OSGi bundle, it is possible to compute a patient’s early warning score based on different scoring configurable models. This makes the system much more configurable and upgradable in terms of computing an early warning score by deploying the latest model. The other benefits that OSGi provides to the HAVEN system are reduced complexity, system scalability, a modular approach, easy deployment and dynamic updates. The system is easily manageable and the components as well as the predictive model can be upgraded without restarting the system.

## Conclusions

We have designed and developed a system to support the identification of patients on wards who would need ICU admission in time to avert the unplanned admission. The system is being evaluated in the context of a single hospital and will need considerable evolution before it becomes ready for widespread adoption.

The solution we have designed uses innovative technologies with the possibility of system scalability and enables simple future upgrades of the predictive model. We are using feedback from our human factors colleagues within the wider HAVEN team to make further enhancements and improvements to the system user interface.

## Availability and requirements

**Project name**: HAVEN (Hospital alerting via electronic noticeboard).

**Project home page**: https://www.ndcn.ox.ac.uk/research/critical-care-research-group-kadoorie-centre/research-studies/hospital-alerting-via-electronic-noticeboard-haven

**Operating system(s)**: Platform independent.

**Programming language**: Java, Scala.

**Other requirements**: Modern web browser.

**License**: Proprietary licence.

**Any restrictions to use by non-academics**: Licence needed.

## Data Availability

Not applicable.
